# The Ankle Joint Range of Motion and Its Effect on Squat Jump Performance with and without Arm Swing in Adolescent Female Volleyball Players

**DOI:** 10.3390/jfmk6010014

**Published:** 2021-02-03

**Authors:** Vassilios Panoutsakopoulos, Mariana C. Kotzamanidou, Georgios Papaiakovou, Iraklis A. Kollias

**Affiliations:** 1 Biomechanics Laboratory, Department of Physical Education and Sports Sciences at Thessaloniki, Aristotle University of Thessaloniki, 54124 Thessaloniki, Greece; gpapaiak@phed.auth.gr (G.P.); hkollias@phed.auth.gr (I.A.K.); 2 Faculty of Health Sciences, Metropolitan College of Thessaloniki, 54624 Thessaloniki, Greece; mkotzamanidou@metropolitan.edu.gr

**Keywords:** biomechanics, vertical jump, force parameters, knee joint, youth training, exercise, video-analysis, flexibility, adolescence

## Abstract

A flexible ankle joint is suggested to be a contributing factor for vertical squat jump (SQJ) performance. The purpose of the study was to investigate the effect of the active (ACT) and passive (PAS) ankle joint range of motion (ROM) on SQJ performed by adolescent female volleyball players. ACT and PAS ankle ROM at knee extension angles of 90, 140, and 180 degrees (180 degrees: full extension) were measured with a video analysis method for 35 female post-pubertal volleyball players (16.3 ± 1.1 yrs, 1.80 ± 0.04 m, 68.8 ± 6.8 kg). Additionally, the players fulfilling previously recommended criteria were assigned to the flexible (*n* = 10) and inflexible (*n* = 8) groups and executed SQJ with and without an arm swing on a force-plate. Results of the 2 × 2 × 3 MANOVA revealed a significant (*p* < 0.05) flexibility type and knee angle effect, as ankle ROM was larger in PAS compared to ACT and as the knee joint progressed from 90 to 180 degrees extension. The 2 × 2 ANOVA revealed a significant (*p* < 0.05) group effect, as flexible players jumped higher in the arm swing SQJ, along with a significant arm swing effect on key SQJ kinetic parameters. In conclusion, a more flexible ankle joint result in improved SQJ performance. Therefore, ankle flexibility training should be implemented in youth volleyball players.

## 1. Introduction

Jumping is considered to be a crucial element in volleyball [1], as it is demonstrated in a variety of skills, such as the spike jump, the block jump, the jump service, and the overhead set with a jump. Due to the importance of jumping in volleyball, vertical jumping tests are custom for the assessment of performance of volleyball players [2,3,4]. The most common tests are the squat jumps (SQJ), the countermovement jumps (CMJ), the drop jumps (DJ), and series of repetitive jumps [3]. Out of these vertical jump tests, the SQJ was found to be highly correlated with the height achieved in a block jump, while the spike jump height, namely the offensive action to hit the ball with a dynamic arm swing during a maximal vertical jump aiming to give the ball high velocity and a steep ball trajectory in the opponents’ court, had a large correlation with CMJ height [5]. However, the dynamic arm swing was found to be a key aspect for jump height in volleyball jumps. This is because the arm swing effects positively the proximal-to-distal coordination during the countermovement, resulting in increased work in the torso and higher angular velocities in the lower limb joints that eventually increase power and ground reaction forces [6]. As in the countermovement jumps, the jump height in SQJ is also larger when an arm swing is used [7,8]. This enhancement in SQJ performance results from the augmented work done by the lower extremities due to the additional load resulted from the mechanical work produced by the shoulder joint during the arm swing [8,9,10].

To generate energy for the impulse to execute a jump, bi-articular muscles have a key role, as the energy generated by proximal muscles appears as mechanical work at a distal joint due to energy transfer facilitated by these muscles leading mechanical energy to be transferred in a proximal to distal joint reversal sequence [11]. This energy is eventually passed through the ankle joint, where its plantar flexion contributes in approximately 22–23% of the take-off velocity [12,13]. The contribution of the ankle joint to a vertical jump depends on the magnitude of the force developed by the ankle plantar flexors, with the gastrocnemius muscle also aiding as a bi-articular muscle to the energy flow, from the differences in their stimulation onset times [14] and from its range of motion, as individuals with poor flexibility jumped less than more flexible individuals [15]. For example, it is unavoidable for an individual with limited ankle dorsiflexion to perform a maximum vertical jump without leaning his trunk forward or lifting his heels off the ground [15]. Research findings suggest that the combined effect of improving the ankle dorsiflexion range of motion and the strength of the muscles acting at the ankle joint could cause improvements in vertical jumping ability [16]. This is probably due to the fact that the ankle dorsiflexors strength is determined by ankle dorsiflexion range of motion [17]. Thus, the ankle joint can be classified as a key joint for the achievement of optimal vertical jumps. This is of importance, since the most common acute injury in volleyball is located mostly in the ankle, namely the ankle sprain [18] and it is the most frequent time loss injury in youth female volleyball players [19]. The importance of flexibility, namely the ability of tissues to achieve the maximum range of movement that is quantitatively measured as joint range of motion (ROM) [20], for injury avoidance and sport-specific performance is well documented [21]. The ankle ROM depends on the ankle dorsiflexion and plantar flexion ability and is evaluated in the sagittal plane, with the knee joint angular position at neutral, in 90° flexion or at maximum knee flexion when the examined person is standing or at the supine position [20]. The range of motion is also measured actively (with muscular contraction only) or passively (movement of the joint caused by external force) [22]. The ankle ROM can be measured with video analysis, which is suggested to be accurate and reliable [23,24,25,26,27,28,29]. However, when performing a jump in a volleyball game, such as a spike jump [6,30], the ankle joint is dorsiflexed when the knee joint angle is in a position in between of those reported above and which are used for the evaluation of ankle ROM.

Vertical jump performance is suggested that is not the single main predicting factor of optimum performance in volleyball [1]. Research has conducted to establish relationships between jumping performance and other factors that contribute to performance [31]. The relationship between flexibility and jumping performance revealed that, for women volleyball players, performance in the vertical jumps was negatively correlated with hip flexibility [32]. However, interventions to improve flexibility in adolescent female volleyball players resulted in enhancements concerning SQJ performance [33] and the effectiveness in volleyball skills [34].

Based on the above, the importance of the ankle ROM in volleyball players is evident. Nevertheless, flexibility is not commonly regarded among the attributes for performance in female volleyball [35]. In addition, to the best of the authors’ knowledge, there is limited evidence about the ankle ROM and its relationship with squat jumping performance in young female volleyball players. Therefore, the first purpose of this study was to evaluate the ankle ROM under the perspective of laterality, knee joint flexion and type of flexibility in young female volleyball players. For this purpose, the ankle ROM was measured for both legs, in an active and passive condition and at three different knee joint angles. The second purpose was to examine possible differences between flexible and inflexible players in the SQJ parameters with and without an arm swing. It was hypothesized that flexible players would perform better than inflexible players and that flexible players would benefit more from the arm swing as the greater mobility of the ankle joint will possibly attribute to an enhanced energy flow for the impulse. The SQJ rather than the countermovement vertical jump was selected to test the hypothesis, aiming to limit possible inter-individual differences caused by altered mechanics due to self-selected downward velocity and lower limb joints flexion in the eccentric phase [36].

## 2. Materials and Methods

### 2.1. Design of the Study

For the examination of the hypotheses of the study, the experimental procedure was conducted in two steps at separate testing sessions. At first, the ankle ROM at knee extension angles of 90°, 140°, and 180° (180° = full extension) was measured for both legs (Experiment 1). Afterwards, the participants that fulfilled previously recommended criteria [37] were assigned to groups with good (“flexible”) and poor (“inflexible”) ankle dorsiflexion and executed SQJ with and without an arm swing on a force-plate (Experiment 2).

### 2.2. Experiment 1: Ankle Range of Motion

#### 2.2.1. Participants

Thirty-five (*n* = 35) female post-pubertal volleyball players (16.3 ± 1.1 yrs, 1.80 ± 0.04 m, 68.8 ± 6.8 kg), members of the respective youth national teams, participated in the study. All participants were identified as adolescents (Stage V) according to the growth assessment technique described by Tanner [38]. The inclusion criteria were that the participants had records of systematic participation in their training program, with no reported injury for a period of six months prior the research. The tests were performed at least 24 h after the last strenuous training session and were part of a wider physical conditioning monitoring program of the national teams that were conducted in accordance with the Declaration of Helsinki and the Research Ethics Code of the Aristotle University of Thessaloniki, after the procedure being approved from the Institutional Research Committee (7233/091197).

#### 2.2.2. Experimental Procedure

**Figure 1:**
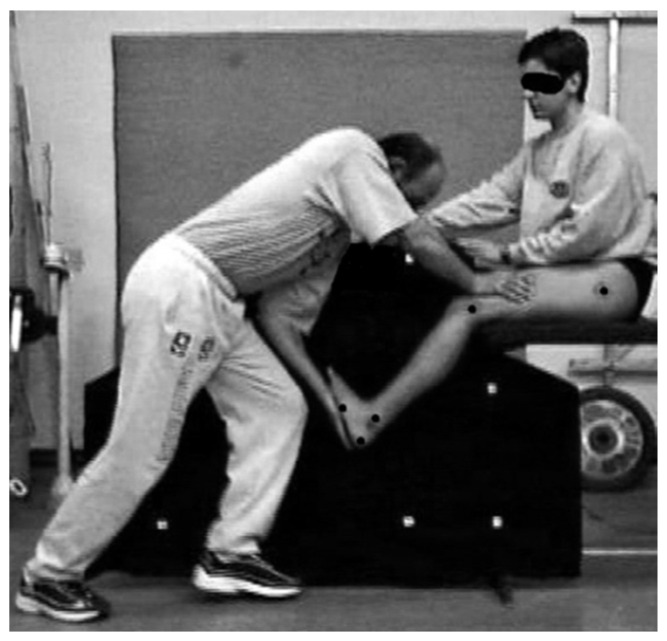
The procedure for the ankle range of motion measurement.

The measurement of ankle ROM was conducted with a video analysis method. The coefficient of reliability for this method is reported to range from 0.942 to 0.975 [15,28]. The participants executed the ankle ROM test barefooted. Custom reflective markers with a diameter of 0.01 m were placed on the greater trochanter, the lateral epicondyle of the femur, the posterior aspect of the calcaneus, and the tuberosity of the 5th metatarsal. These anatomical points were marked as they represent the hip, knee, ankle and metatarsal joints. In addition, a marker was attached on the lateral malleolus (Figure 1).

After the placement of the markers, the participants sat, with their torso in an upright position, on an examination bed. The knee joint was at the edge of the bed, allowing the shank to swing freely. Then, the participants were fixed on the bed and were asked to plantar flex at their maximum capability while slowly swinging their shank from the vertical to the horizontal position, and vice versa. The same procedure was repeated with a dorsiflexion. These assessments comprised the active flexibility measures (ACT). For the assessment of the passive flexibility of the ankle joint (PAS), the same experienced researcher applied force on the dorsal surface of the foot to cause a plantar flexion of the ankle joint until the maximum ROM was achieved and a feeling of discomfort was reported from the participant. Then, the same as above shank swing was executed. Afterwards, the force was applied on the plantar surface of the foot and the shank motion was repeated.

The above described procedure was conducted in a randomized order for both legs in an examination room with an ambient temperature (22–23 °C) and without the participants to perform any kind of warm-up. For the measurement of the ankle ROM with the 2D-DLT video analysis method [39], the plane where the movement was performed was calibrated placing a 1.25 m × 1.25 m calibration frame containing 10 reference markers prior the measurement. The calibration procedure was conducted to establish that the axis of the camera was vertical to the plane of movement and for the extraction of the position of the markers in a two-dimensional Cartesian coordinate system.

#### 2.2.3. Data Acquisition and Analysis

The experimental procedure was recorded with a Panasonic NV-MS4E (Matsushita Electric Industrial Company, Osaka, Japan) from a distance of 4 m. The camera was fixed on a tripod at a height of 1.2 m and was operating with a sampling frequency of 25 fps. The captured motion was then projected field by field using a Citizen 30PC-1EA projector (Japan CBM Corp., Tokyo, Japan) on a COMPLOT 7000 digitizer (Mayline Company Inc., Sheboygan, WI, USA) and the coordinates of the markers were stored online in a personal computer. Thus, the knee and ankle joint angles were estimated using the extracted coordinates and basic trigonometric equations as follows:

- ankle angle (θank): the angle formed by the shank (i.e., the line defined by the lateral epicondyle of the femur and the posterior surface of the calcaneus) and the foot (i.e., the line defined by the tuberosity of the 5th metatarsal and the lateral malleolus), where θank = 90° meant that the shank was perpendicular to the foot. Ankle dorsiflexion was noted when θank < 90° and ankle plantar flexion when θank > 90°;
- knee angle (θknee): the angle formed from the shank and the thigh (i.e., the line defined by the lateral epicondyle of the femur and the greater trochanter).

Initially, θknee was defined with an approximate error of ± 1.5°, as revealed in a test-retest comparison of 10% of the fields that were re-digitized for this evaluation procedure. θank was calculated afterwards. The ACT and PAS ankle ROM was calculated using the θank recorded for the dorsi- and plantar-flexion when θknee was at 90°, 140°, and 180°, where 180° represents a fully extended knee joint. All the above measurements were conducted using a custom made software (ANGLES software, ©: Iraklis A. Kollias, Biomechanics Laboratory, Aristotle University of Thessaloniki, Thessaloniki, Greece).

#### 2.2.4. Statistical Analysis

Descriptive statistics for the examined parameters are presented as mean ± standard deviation. A 2 (leg; Left, Right) × 2 (condition; ACT, PAS) × 3 (θknee; 90°, 140°, 180°) MANOVA with repeated measures on the last factor after Bonferroni adjustments was conducted to test the effect of laterality, type of assessment, and knee angle on ankle ROM. Significant differences were followed up with simple contrasts. Effect sizes were checked using the partial eta-squared statistic (*η_p_*^2^), with values of above 0.01, 0.06, and 0.14 being considered as small, medium, and large, respectively [40]. All statistical analyses were conducted using the IBM SPSS Statistics v.25 software (International Business Machines Corp., Armonk, NY, USA), with the level of significance set at *a* = 0.05.

### 2.3. Experiment 2: Vertical Squat Jump Performance

#### 2.3.1. Participants

After the completion of Experiment 1, the participants that fulfilled the criteria set in previous studies [37] were assigned to the flexible (FLG) and inflexible (IFG) group. The inclusion criterion for the FLG group was the PAS dorsiflexion θank to be less than 59.8° when θknee was 90°. The respective criterion for IFG was that the PAS dorsiflexion θank should be higher than 71.8°. These criteria were adopted on the basis of past research findings in a large cohort of female physical education students, where the frequency distribution analysis identified the previously mentioned values to indicate individuals as “flexible” (7.5th percentile) and “inflexible” (92.5th percentile), respectively [37]. Another inclusion criterion was that the interlimb difference of PAS dorsiflexion θank should not be more than 10.0°. Thus, 10 players formed the FLG (16.6 ± 1.2 yrs, 1.80 ± 0.04 m, 67.8 ± 6.8 kg), whereas 8 players were considered in the IFG (15.7 ± 0.7 yrs, 1.79 ± 0.06 m, 70.4 ± 8.0 kg).

#### 2.3.2. Experimental Procedure

Participants performed a typical 20-min warm-up, consisting of 10 min cycling on an 817E Monark Exercise Cycle (Exercise AB, Vansbro, Sweden) at constant velocity of 5.5 m·s^−1^ with 0 W load, followed by a 10 min dynamic stretching program with movements that gradually engaged the joints to move in almost full range of motion. A section of self-administered squatting exercises and sub-maximal vertical jumps for familiarization was performed afterwards. Before the testing session, the participants were instructed about the execution of the SQJ with (AS) and without the arm swing (NAS). At the starting position for the execution of the SQJ-NAS, the arms were placed on the hips, the feet were in full contact with the force-plate and θknee was approximately 90°. The arms were placed on the hips throughout the jump, the flight and the landing. The validity of the SQJ-NAS was checked as described in previous research [41]. For the SQJ-AS, the arms were kept hanging vertically, parallel at the side of the body, and were then swung upwards during the impulse. In total, three SQJ-AS and three SQJ-NAS were performed in a random counterbalanced order.

All jumps were performed barefooted. A minimum of a 60 s resting period was allowed between trials to avoid fatigue. The instruction given to the participants was to “jump as high and as fast as possible without a downward movement”. The best attempt of the three trials, using as criterion the maximum jump height achieved (Hjump), was selected for further analysis.

#### 2.3.3. Data Acquisition and Analysis

Ground reaction forces (GRF) were recorded with an AMTI OR6-5-1 force plate (AMTI, Newton, MA, USA) connected on line with a Pentium II PC in which GRF recordings were stored after being converted to digital using a PC-LabCard PCL-812PG (Advantech Co., Taipei, Taiwan) 12-bit analogue-to-digital converter. Data acquisition was set to a nominal sampling frequency of 500 Hz. The signal was digitally smoothed using a 2nd order low-pass Butterworth filter, with cut-off frequency set at 8 Hz.

Hjump was calculated based on the body center of mass (BCM) vertical take-off velocity (Vy) computed after the integration of the vertical GRF. The kinetic parameters of the SQJ were extracted based on the force-time history and the mass of the participants. In detail, besides the maximum vertical GRF (FZmax), the maximum rate of force development (RFDmax) was extracted as the peak value of the first-time derivative of the vertical GRF, the maximum power output (Pmax) was the peak value of the multiplication of the vertical GRF with vertical BCM velocity. The integration of vertical BCM velocity revealed the vertical BCM displacement from the initial starting position to the instant of take-off (Sto). The mechanical work (Wmax) was defined as the peak value computed by multiplying vertical GRF with the vertical BCM displacement. In addition, temporal parameters, such as the total duration of the impulse (tC) and the time to achieve the peak vertical GRF (tFz), were included in the analysis. Finally, the efficiency of AS in Hjump (AS_efficiency_) was assessed as shown in Equation (1):

(1) $$AS_{efficiency}=\frac{\left(Hjump_{AS}-Hjump_{NAS}\right)}{Hjump_{NAS}}\times 100.$$

#### 2.3.4. Statistical Analysis

Descriptive statistics for the examined parameters are presented as mean ± standard deviation. Normality of distribution and the equality of variance were assessed using the Shapiro-Wilk test (*p* > 0.05) and the Levene’s test (*p* > 0.05), respectively. According to the results of the Shapiro-Wilk test, an Independent Samples *T*-test was run to check the difference concerning the efficiency of AS in Hjump between FLG and IFG. The effect size was checked using the Hedges’ *g*, with values of <0.2, <0.5, <0.8, and ≥0.8 being considered as trivial, small, medium, and large, respectively [42]. A 2 (flexibility; FLG, IFG) × 2 (arm swing: NAS, AS) repeated measures ANOVA with Bonferroni adjustment was carried out to compare the main effects of flexibility and arm swing and the interaction effect between flexibility and arm swing on the kinetic and temporal parameters of the SQJ. Significant differences were followed up with pairwise comparisons. Effect sizes for this procedure were checked using the partial eta-squared statistic (*η_p_*^2^), with values of above 0.01, 0.06, and 0.14 being considered as small, medium, and large, respectively [40]. All statistical analyses were conducted using the IBM SPSS Statistics v.25 software (International Business Machines Corp., Armonk, NY, USA), with the level of significance set at *a* = 0.05.

## 3. Results

### 3.1. Experiment 1: Ankle Range of Motion

The results of the measurements of the ankle ROM are presented in Table 1. All ROM measurements in the PAS condition were approximately 12 to 18° higher compared to the respective ACT condition, resulting in a significant type of flexibility assessment effect (*F*_1,130_ = 296.359, *p* < 0.001, *η_p_*^2^ = 0.820; large effect).

It was also observed that the ankle ROM was progressively decreased as θknee changed from 90° flexion to full extension, presenting a significant knee joint angle effect (*F*_2,130_ = 184.984, *p* < 0.001, *η_p_*^2^ = 0.740; large effect). Finally, the interlimb differences ranged from 0.8 to 6.6°, and no significant lateral effect was observed (*F*_1,65_ = 0.216, *p* = 0.644, *η_p_*^2^ = 0.003).

### 3.2. Experiment 2: Vertical Squat Jump Performance

Based on the outcome of Experiment 1, ten players with θank less than 59.8° formed FLG (θank = 55.4 ± 3.4°), and eight players with θank over 71.8° formed IFG (θank = 74.3 ± 4.7°). The results for their SQJ tests are presented in Table 2. Compared to SQJ-NAS, Vy at SQJ-AS take-off increased by 0.03 ± 0.06 m/s in FLG and 0.06 ± 0.09 m/s in IFG, respectively. A non-significant between group difference (*t*_1,16_ = 0.933, *p* = 0.365, *g* = 0.293) concerning the efficiency of AS in Hjump was observed, as it was found to be 2.6 ± 5.4% and 0.4 ± 8.9% for FLG and IFG, respectively.

On average, FLG jumped 3.8 cm higher than IFG in the SQJ-AS (Table 2). This represented a large effect size significant main flexibility effect (*F*_1,32_ = 8.840, *p* = 0.006, *η_p_*^2^ = 0.216) on Hjump. Significant large effect size main flexibility effects were also observed for Pmax (*F*_1,32_ = 6.239, *p* = 0.018, *η_p_*^2^ = 0.163) and Wmax (*F*_1,32_ = 6.292, *p* = 0.017, *η_p_*^2^ = 0.164). Finally, significant medium effect size main flexibility effect was evident for tFz (*F*_1,32_ = 4.685, *p* = 0.038, *η_p_*^2^ = 0.128).

As for the effect of the arm swing, results revealed that, on average, FLG jumped 0.9 cm higher in the SQJ-AS than in the SQJ-NAS. On the opposite, the increase in Hjump for IFG was on average just 0.1 cm higher in the SQJ-AS compared to the SQJ-NAS. Thus, there was no significant main arm swing effect on Hjump (*F*_1,32_ = 0.182, *p* = 0.672, *η_p_*^2^ = 0.006). On the contrary, significant large effect size main arm swing effects were observed for FZmax (*F*_1,32_ = 12.331, *p* = 0.001, *η_p_*^2^ = 0.278), RFDmax (*F*_1,32_ = 7.277, *p* = 0.011, *η_p_*^2^ = 0.185), Pmax (*F*_1,32_ = 7.237, *p* = 0.011, *η_p_*^2^ = 0.184), Wmax (*F*_1,32_ = 7.640, *p* = 0.009, *η_p_*^2^ = 0.193), and Sto (*F*_1,32_ = 6.143, *p* = 0.019, *η_p_*^2^ = 0.161).

**Figure 2:**
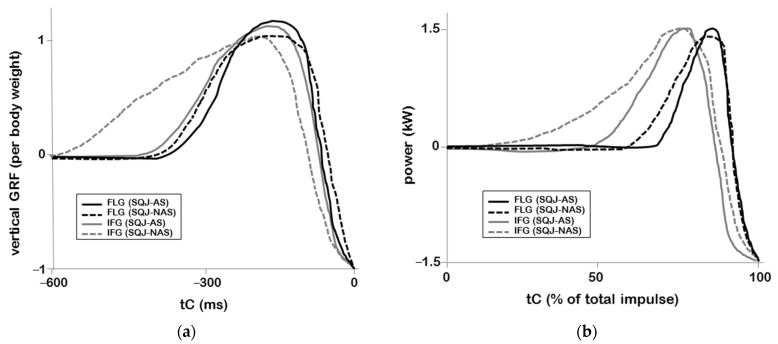
Representative individual time history curves for (**a**) vertical ground reaction forces (tC = 0 indicates the instant of take-off); (**b**) power output. Dashed lines present the squat jump test without an arm swing (SQJ-NAS) and solid lines the squat jump test with an arm swing (SQJ-AS). Black lines depict a flexible player (FLG) and gray lines an inflexible player (IFG).

Finally, there was no significant interaction among the two vertical jump tests and the two groups for the examined parameters. As presented in Figure 2, a similar pattern between FLG and IFG was identified for vertical GRF and especially for the power output. A detailed examination of the representative individual curves for a selected FLG and IFG player reveals that the vertical GRF was higher in the SQJ-AS compared to the SQJ-NAS (Figure 2a). Despite the fact that power had an almost identical peak value for both SQJ-NAS and SQJ-AS, the slope of the power output curve was steeper in the FLG compared to the IFG player (Figure 2b).

## 4. Discussion

Results of the ankle joint range of motion revealed significant type of flexibility and knee joint angle effects, with no differences concerning the interlimb comparison. It was found that passive range of motion was significantly larger than the active condition. In addition, significantly larger ankle range of motion was recorded when the knee joint was progressively flexed from its full extension. As for the squat jump tests, significant flexibility and arm swing effects were observed, but the hypotheses of the study were partly confirmed. The flexible players did perform better in the squat jump with an arm swing compared to the inflexible players, but the effectiveness of using the arm swing, although larger in the flexible players, was not significant different between the examined groups.

The passive ankle range of motion when the knee joint was at 40° knee flexion was on average about 82°. This value is larger compared to previous findings for female handball players [43] and professional male soccer players [28]. Previous research [44] found that the active ankle range of motion, when the knee joint is 90° flexed, is approximately 70°, which is also confirmed in the present study. Nevertheless, the trend that the ankle range of motion decreases when the knee joint extends is in agreement with past research [45]. In addition, the present findings regarding the differences between active and passive assessment of the ankle joint range of motion are also in agreement with the literature [43,46]. The distinct reduction of the passive ankle ROM when the knee was fully extended compared to the other flexed knee joint positions can be attributed to the previous finding that the largest changes of the architecture of the gastrocnemius muscle are evident in the range of approximately 35° knee flexion to its full extension [47]. This eventually leads to differentiations of the musculotendinous junction [48,49]. In specific, the relative length of the musculotendinous unit and its moment of inertia increase when the knee joint is extended [50]. In addition, the ankle dorsiflexion is affected in a greater magnitude during knee extension rather than knee flexion, as this extension is influenced by the bi-articularity of the gastrocnemius muscle [51]. Furthermore, when the knee is extended, the gastrocnemius muscle is stretched and thus to a reduction of the ankle dorsiflexion when the joint is not loaded. Thus, during knee flexion, the gastrocnemius muscle is relaxed due to its attachment on the femur and thus it is not that resistant to the non-weight bearing ankle dorsiflexion [51]. The absence of significant lateral differences concerning the ankle ROM confirms previous observations in various sports [28,43,52]. However, the non-significant trend noted for larger values of passive ROM for the left ankle, along with lower active ankle ROM compared to the right leg, can be attributed to the sport specific demands of the volleyball jumps that are different for each lower limb. For example, during the execution of the preparation steps in the spike jump, female players differentiate the planting and the movement pattern of the lower extremities [6]. This interlimb differentiation in loading may resulted in the observed trend in ankle ROM. This finding adds to the current bias in the literature concerning the fact that different lower-limb tests produce different results concerning inter-limb asymmetry indexes [53,54].

The data of the present study showed that the height achieved in the SQJ without an arm swing, despite being on average 3 cm higher in the flexible group, was not significant different between groups, a fact that was also observed in the past for female physical education students [37]. Lower squat jump height was reported when the ankle range of motion is restricted during a squat jump [55]. In the case of inflexible or reduced ankle mobility, the possible reduced contribution of the energy transfer through the biarticular gastrocnemius muscle is counterbalanced with adaptations, such as enhanced mechanical output at the knee [55] or a larger mobility of the hip joint and the torso [56]. On the opposite end, significant differences were revealed for the SQJ with an arm swing, as the efficiency of the arm swing was just 0.4% for the inflexible players. Nevertheless, previous research revealed low efficiency of the use of the arm swing in females compared to males, possibly due to the greater upper body strength of men [7]. Non-significant larger maximum vertical ground reaction forces and rate of force development values were observed for the inflexible group. This is suggested to lead to greater peak angular accelerations that eventually result in an enhancement in vertical body center of mass velocity [57]. However, in the present study, this did not contribute to larger take-off velocity in the inflexible group, perhaps due to smaller range of motion caused by the reduced ankle joint dorsiflexion [56,58].

A significant flexibility and arm swing effect concerning the power and mechanical work output was noted in the tested volleyball players. This is in agreement with previous findings regarding the factors that differentiate SQJ performance among groups of young female athletes, which are the whole body peak mechanical power output and the force/time structure of the jump [41]. This fact is important in volleyball, as the force/time structure of the biomechanical parameters that optimize the jump must meet the combined demand for the restrictions of time and constrains in space for a maximization of the propulsive impulse [10,59]. The present results also support previous findings that the use of the arm swing increases performance in the SQJ as the jump height in the arm swing squat jump is suggested to be related with increased force application, power production, and work output compared to no arm swing vertical jumps [7,8,9,10,60]. As presented in past research [57], the time to achieve maximum vertical ground reaction force was less in the arm swing squat jump than the no arm swing test, but it was evident only in the flexible group. The opposite was observed for the inflexible group. Concluding, it is suggested that the enhancement of jumping performance is achieved by maximizing the capabilities of the lower limb neuromuscular system concerning its power output and by optimizing its force-velocity mechanical profile [61].

The active and/or passive stretching of the gastrocnemius muscle is the main factor to control the ankle dorsi flexion [62], which, in turn, is suggested to be the determining factor for the optimization of the vertical squat jump performance [56]. The present findings can be interpreted under the perspective that the alteration of the angular position of the examined joints result in changes in the length of the gastrocnemius muscle and eventually to the function of its musculotendinous unit [47,50,63,64] and the neuromechanical function of the ankle joint flexors and extensors [65,66,67,68,69,70]. It is suggested that these modifications due to the different ankle angle cause alterations in the force application capabilities of the shank muscles, as well as in the transfer of the power [62,71,72,73,74,75,76,77]. If constrains exist when executing a vertical squat jump, either in the form of ankle mobility reduction [55] or the demand for a full feet contact [78], then several adaptations occur as the muscle activation patterns are adjusted for the maximization of the jump height from the altered initial posture [79]. Despite the fact that different initial postures were found not to affect vertical jumping performance [80], inflexible athletes achieve better vertical jump performance when the jump is executed with a large forward lean of the body [56,78]. However, this body posture is suggested to cause, besides the execution of the jump with less balance during the propulsive phase, additional loading at the Achilles tendon and the spine [37,78]. In addition, enhanced output at the knee joint is evident [55]. In the case of the squat jump with an arm swing, simulation studies have shown that larger amount of work is produced by the gastrocnemius muscle compared to the no arm swing squat jump [8]. These facts combined contribute to findings that the reduced ankle dorsiflexion range might increase the risk of patellar tendon injury among volleyball players [81]. Thus, the importance of gastrocnemius stretching is emphasized concerning the prevention of ankle sprain in volleyball players, and it is recommended to be involved in the preventive training programs in volleyball players [82].

The body posture, combined with joint kinematics, joint torque, and muscle activation patterns, could possibly add content to the interpretation of the present findings. Another possible limitation is the lack to measure the strength of the upper arm and shoulder area muscles and to examine their possible effect on the reported results. Finally, the study focused on the effect of the muscular element involved in the ankle joint mobility and not on constrains imposed by the skeletal system, specifically examining the effect of the bone structure and deformities of the ankle joint and the feet. Nevertheless, the results of the study demonstrated that individuals with larger ankle dorsiflexion angle can utilize more efficiently the additional work provided by the arm swing in the vertical squat jump compared to individuals with less flexible ankle joint.

## 5. Conclusions

The vertical squat jump kinetic parameters are differentiated when an arm swing is used. The arm swing is effectively used by adolescent female volleyball players with large ankle dorsiflexion, as a larger gain in performance was presented. On the opposite end, adolescent female volleyball players with limited ankle dorsiflexion were not able to increase their vertical squat jump height compared to the no arm condition. In addition, significantly larger ankle range of motion was recorded when the knee joint was progressively flexed from its full extension. The modifications concerning the mobility of the ankle joint caused by different knee joint angles should be considered by coaches and practitioners with regard the demands to execute technique elements where a combined knee and ankle joint movement is essential. Alterations in the relative position of the ankle and knee joints can lead to differences in tissue loading that could cause, besides the incident of an injury, adaptations in a different manner than those that the training program is aiming to develop. Thus, flexibility tests should be implemented in a regular basis. In addition, the training programs should aim to improve the ankle range of motion, especially the ankle dorsiflexion, for the enhancement of the effectiveness of the use of the arm swing in vertical jumping that is essential in volleyball. Regarding the interlimb effect in the range of motion measurements, future research should also take into consideration the interlimb comparison in terms of dominant versus non-dominant leg or with respect to the contralateral and ipsilateral leg referred to the preferred arm to execute the spike jump. Along with the interlimb effect, the differences in the examined parameters concerning the player position could also be of interest to investigate.

## Figures and Tables

**Table 1:** Results for the ankle joint range of motion (ROM) measurements (*n* = 35).

	θknee = 90°	θknee = 140°	θknee = 180°
ROM Measurement	Mean ± SD	Mean ± SD	Mean ± SD
Right leg			
ACT (deg)	74.4 ± 9.5	67.7 ± 9.5 ^a^	62.9 ± 9.7 ^a,b^
PAS (deg)	87.1 ± 9.5 *	79.8 ± 7.9 *^,a^	75.7 ± 7.8 *^,a,b^
Left leg			
ACT (deg)	67.8 ± 10.8	66.9 ± 11.5	61.3 ± 11.1 ^a,b^
PAS (deg)	90.9 ± 9.7 *	85.1 ± 9.2 *^,a^	78.0 ± 8.1 *^,a,b^

^a^: significantly different compared to θknee = 90° (*p* < 0.05); ^b^: significantly different compared to θknee = 140° (*p* < 0.05); *: significantly different compared to ACT (*p* < 0.05); θknee: knee joint angle; ACT: active flexibility range of motion test; PAS: passive flexibility range of motion test.

**Table 2:** Results of the examined parameters for the vertical squat jump (SQJ) with (AS) and without (NAS) the use of an arm swing executed by the players of the flexible (FLG, *n* = 10) and inflexible (IFG, *n* = 8) groups.

Parameter	SQJ	FLG (*n* = 10)	IFG (*n* = 8)	Flexibility		Arm Swing		Interaction	
		Mean ± SD	Mean ± SD	*p*	*η_p_* ^2^	*p*	*η_p_* ^2^	*p*	*η_p_* ^2^
Hjump	NAS	22.3 ± 3.6	19.3 ± 2.1	0.006 ^f^	0.216	0.672	0.006	0.696	0.005
(cm)	AS	23.2 ± 4.5	19.4 ± 2.4 *						
FZmax	NAS	1.8 ± 0.4	2.0 ± 0.2	0.202	0.05	0.001 ^s^	0.278	0.325	0.03
(N/kg)	AS	2.2 ± 0.2 ^#^	2.3 ± 0.2						
RFDmax	NAS	6.0 ± 1.8	6.4 ± 2.9	0.682	0.005	0.011 ^s^	0.185	0.993	<0.001
(kN/sec)	AS	8.8 ± 3.8	9.2 ± 3.4						
Pmax	NAS	21.3 ± 2.8	18.9 ± 2.0	0.018 ^f^	0.163	0.011 ^s^	0.184	0.787	0.002
(W/kg)	AS	24.4 ± 4.2 ^#^	21.5 ± 3.0						
Wmax	NAS	2.3 ± 0.6	1.9 ± 0.4	0.017 ^f^	0.164	0.009 ^s^	0.193	0.967	<0.001
(J/kg)	AS	1.9 ± 0.5	1.5 ± 0.4						
Sto	NAS	52.3 ± 6.0	48.6 ± 6.5	0.051	0.114	0.019 ^s^	0.161	0.851	0.001
(cm)	AS	47.7 ± 6.2	43.2 ± 5.0						
tC	NAS	788 ± 125	720 ± 173	0.281	0.036	0.825	0.002	0.758	0.003
(ms)	AS	762 ± 139	724 ± 144						
tFz	NAS	631 ± 129	523 ± 165	0.038 ^f^	0.128	0.469	0.017	0.747	0.003
(ms)	AS	585 ± 122	505 ± 93						

*: significantly different compared to FLG (*p* < 0.05); ^#^: significantly different compared to NAS (*p* < 0.05); ^f^: significant flexibility effect; ^s^: significant arm swing effect; Hjump: jump height; FZmax: maximum vertical ground reaction force; RFDmax: maximum rate of force development; Pmax: peak power output; Wmax: peak mechanical work; Sto: vertical body center of mass displacement during the impulse; tC: total duration of the impulse; tFz: time to achieve FZmax.
